# Brace treatment for patients with Scheuermann's disease - a review of the literature and first experiences with a new brace design

**DOI:** 10.1186/1748-7161-4-22

**Published:** 2009-09-29

**Authors:** Hans-Rudolf Weiss, Deborah Turnbull, Silvia Bohr

**Affiliations:** 1 Orthopedic Rehabilitation Services, Alzeyer Str. 23, D-55457 Gensingen, Germany; 2 ERS, Ealing Hospital, Uxbridge Road, Southall, UB1 3HW, London, UK

## Abstract

**Background:**

In contemporary literature few have written in detail on the in-brace correction effects of braces used for the treatment of hyperkyphosis. Bradford et al. found their attempts effective, treating Scheuermann's kyphosis with Milwaukee braces, but their report did not specifically focus on in-brace corrections. White and Panjabi's research attempted to correct a curvature of > 50° with the help of distraction forces, but consequently led to a reduction in patient comfort in the application of the Milwaukee brace. In Germany they avoid this by utitlising braces to treat hyperkyphosis that use transverse correction forces instead of distraction forces. Further efforts to reduce brace material have resulted in a special bracing design called kyphologic™ brace. The aim of this review is to present appropriate research to collect and evaluate possible in-brace corrections which have been achieved with brace treatment for hyperkyphosis. This paper introduces new methods of bracing and compares the results of these with other successful bracing concepts.

**Materials and methods:**

56 adolescents with the diagnosis of thoracic Scheuermann's hyperkyphosis or a thoracic idiopathic hyperkyphosis (22 girls and 34 boys) with an average age of 14 years (12-17 yrs.) were treated with the kyphologic™ brace between May 2007 and December 2008. The average Stagnara angle was 55,6° (43-80). In-brace correction was recorded and compared to the initial angle using the t-test.

**Results:**

The average Stagnara angle in the brace was 39°. The average in-brace correction was 16.5° (1-40°). The verage percentage of in-brace correction compared to the initial value was 36%. The differences were significant in the t-test (t = 5.31, p < 0,001). To make these results comparable to other studies, the kyphosis angle of 25° was set to 0 for our sample in order to achieve a norm value adapted (NVA) percentage of in-brace correction. By doing this a correction of 54.1% was achieved. There was no correlation between the percentage of in-brace correction and the age of the patient, but a highly significant correlation between percentage of in-brace correction and the initial Stagnara angle.

**Discussion:**

If we assume that outcome of brace treatment positively correlates with in-brace correction, the treatment should be initiated before the curvature angle exceeds 50 - 55° in a growing adolescent. In scoliosis bracing, if the average in-brace correction equals > 15°, then it is predicted that the result will lead to a final correction. Applying this to hyperkyphosis patients, the average in-brace correction with this brace was also > 15°. We therefore estimated to achieve a favourable outcome using this brace type (once compliance was attained) especially when comparing the correction effects achieved with this new approach to the correction effects reported upon using the Milwaukee brace. The latter brace has been shown to lead to beneficial outcomes in long-term studies with comparable in-brace corrections.

**Conclusion:**

Conservative treatment of Scheuermann's hyperkyphosis in international literature is generally regarded as an effective treatment approach. Physiotherapy and bracing are the first-line treatments for this condition.

An average in-brace correction of > 15° as was achieved using the kyphologic™ brace predicts a favourable outcome.

The kyphologic™ brace leads to in-brace corrections comparable to those of the Milwaukee brace, which has previously been shown to provide beneficial outcome in the long-term.

A prospective follow-up study seems desirable before final conclusions can be drawn.

Future studies should focus more on thoracolumbar and lumbar curve patterns, because these patterns may predict chronic low back pain in adulthood with reduced quality of life of the patients and high costs with respect to medical care and occupational sickness leave.

Surgery according to international literature is rarely necessary in this condition.

## Background

Scheuermann's disease initially was described as a rigid kyphosis associated with wedged vertebral bodies occurring in late childhood [1]. Scheuermann's disease has been of significant orthopaedic interest in the past, as it may be painful during its relative acute phase and more importantly, because it may cause significant trunk deformity that can progress. Sorensen subsequently described specific criteria for diagnosis in 1964 [2], in that three adjacent vertebrae must be wedged at least 5° each.

**Figure 1:**
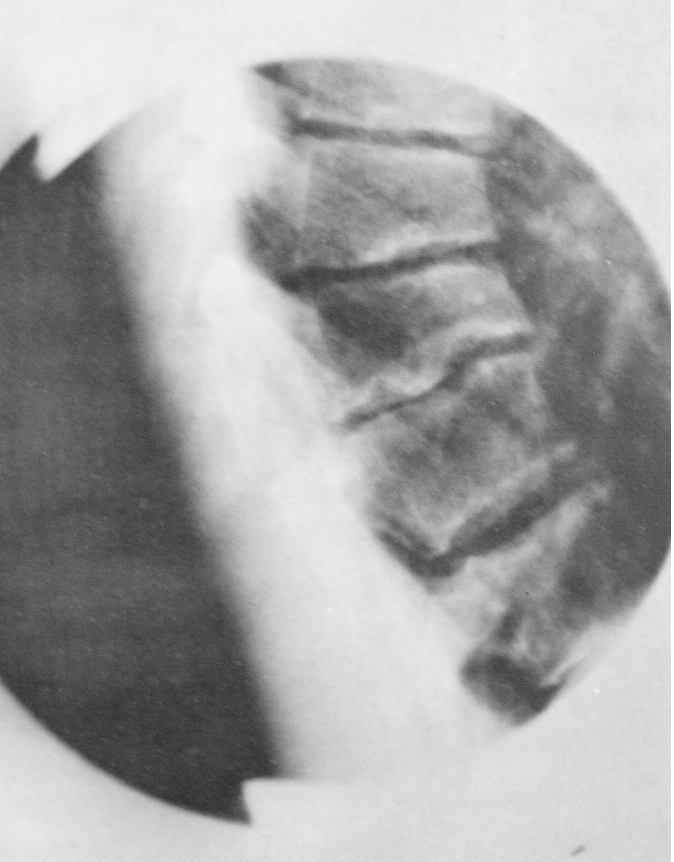
**Scheuermann signs on a lateral X-ray**. Characteristic radiographic findings in a patient with Scheuermann's disease in the late stage (kyphosis, wedging of vertebral bodies, endplate irregularities, Schmorl's nodes).

Others articles have used different criteria. These include increased thoracic kyphosis, disc space narrowing and irregular endplates associated with a single-wedged vertebra [3,4], a kyphosis of greater than 45° with two or more wedged vertebra [5], or 'characteristic' radiographic findings (kyphosis, wedging of vertebral bodies, endplate irregularities, Schmorl's nodes, see Figure 1.) [6,7].

Wenger and Frick [8] in 1999 published an extensive review on this condition, but when looking into recent Pub Med listings, the condition of Scheuermann's kyphosis in the past 10 years seems to stimulate less scientific interest. There are some points of discrepency upon the definition of the pathological deviations of normal and sagittal spinal alignment [8]. Unlike scoliosis, where any significant lateral deviation in the coronal plane is abnormal, the sagittal alignment of the spine has a normal range of thoracic kyphosis. The Scoliosis Research Society has defined this range as being from 20° to 40° in the growing adolescent [9-11]. In a study of 316 healthy subjects with ages ranging from 2 to 27 years, the upper limit of normal kyphosis was noted to be 45°. It was also noted that the average thoracic kyphosis increases with age from 20° in childhood, to 25° in adolescents, to 40° in adults [12]. The lack of a consistent definition of Scheuermann's kyphosis in the literature makes it difficult to compare studies as the inclusion criteria may differ, thus making the distinction between the spectrum of upper normal thoracic kyphosis, severe adolescent roundback deformity, and Scheuermann's disease almost impossible [8].

**Figure 2:**
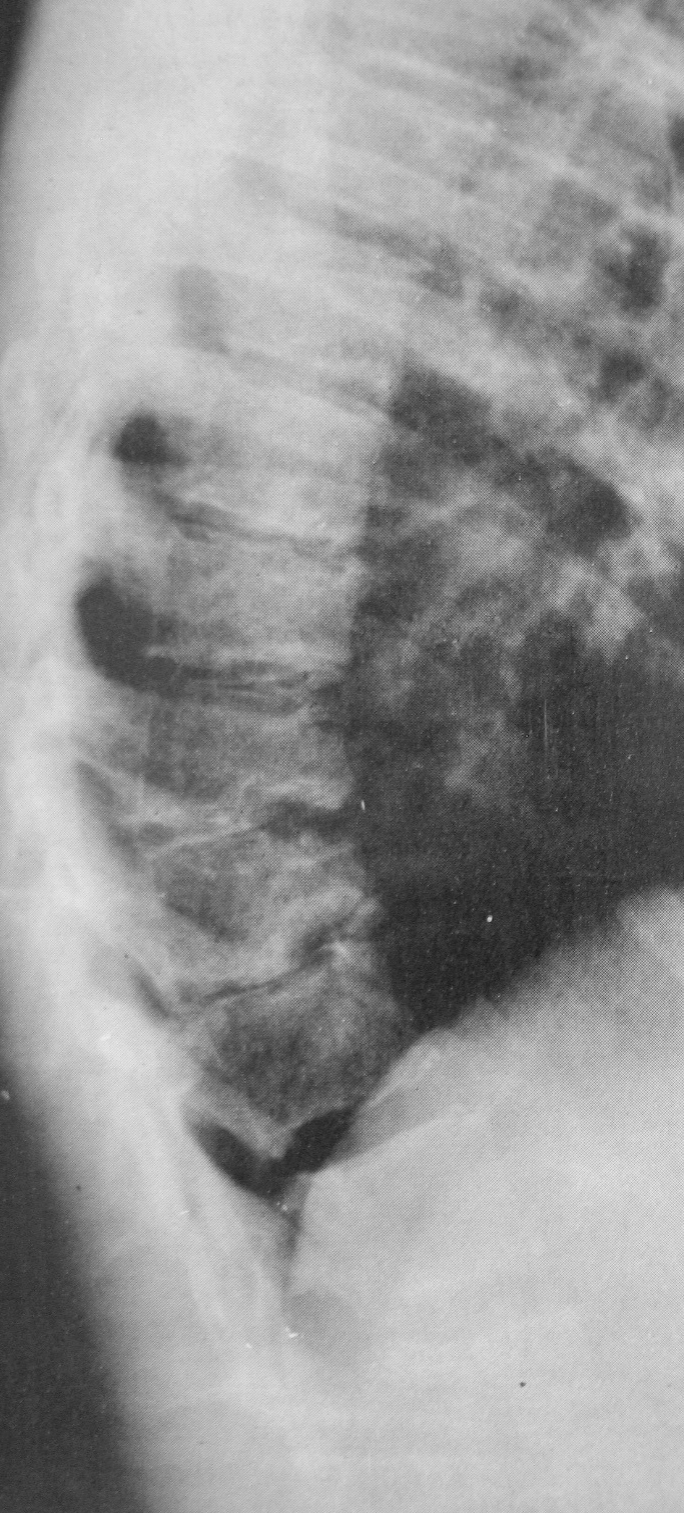
**Scheuermann signs on a lateral X-ray in the thoracolumbar region**. The Schmorl's nodes and endplate irregularity may be so severe that lumbar Scheuermann's disease can be confused with infection, tumor, or other conditions [8].

Little is written on the subject of the lumbar or thoracolumbar patterns of Scheuermann's disease. The Schmorl's nodes and endplate irregularity may be so severe that the lower lumbar Scheuermann's disease can be confused with infection, tumor, or other conditions [8] (Figure 2.). The etiology of lumbar Scheuermann's kyphosis is unknown, but strong associations with repetitive activities involving axial loading of the immature spine favour a mechanical cause [8]. Although the radiographic appearance may be similar, lumbar Scheuermann's kyphosis is regarded as a different entity than thoracic Scheuermann's kyphosis [8]. Unlike classic thoracic Scheuermann kyphosis, the treatment of lumbar Scheuermann's disease was not controversial in 1999 [8], as its course has been regarded as being non-progressive and its symptoms have been regarded to resolve with rest, activity modification and time [13,14].

This loss of lordosis in this area of the lumbar or thoracolumbar spine means that Scheuermann's disease can be one of the predictors of developing chronic low back pain in adulthood:

Loss of lumbar lordosis correlates well with the incidence of chronic low back pain in adulthood [15,16]. Sedentary lifestyle contributes to loss of lumbar lordosis as well as scoliosis and thoracolumbar or lumbar kyphosis [17]. It is necessary to recognise that the severity of symptoms in patients with back pain, as they increase in a linear fashion with progressive sagittal imbalance. The results of these studies also show that hyperkyphosis is more favourable in the upper thoracic region but very poorly tolerated in the lumbar spine [15-17]. As it has been shown, lumbar re-lordosation stabilises the spine with respect to lateral deformity [18], so we may assume that lumbar de-lordosation or lumbar kyphosis destabilises the spine and can lead to chronic low back pain [19]. Ten years after this review by Wenger and Frick [8], lumbar Scheuermann's disease ought to have been investigated specifically, focussing upon the prevention of chronic low back pain in adulthood.

According to Wenger and Frick [8] the incidence of Scheuermann's disease has been estimated at 1 to 8% of the population [2,20]. The typical presentation is in the late juvenile age period from 8 to 12 years, with the more severe fixed form commonly appearing between age 12 and 16 years. Patients with thoracic roundback, who have classic type I Scheuermann's disease, may have pain in the thoracic spine area, but more frequently present because of patient and parental concerns related to trunk deformity. The gender prevalence of Scheuermann's kyphosis is difficult to determine from the literature, and may be related to how Scheuermann's kyphosis is defined. In general, males and females are involved with equal frequency [9], although the reported ratios have varied widely [8].

**Figure 3:**
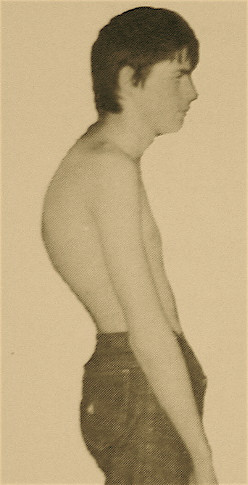
**Lateral view of a patient with severe Scheuermann disease**. Patients with Scheuermann's kyphosis may have an angular thoracic kyphosis, often with accompanying compensatory lumbar lordosis and increased cervical lordosis. The position of the head is often in forward protrusion (so called gooseneck), and the shoulders are often positioned anteriorly as well.

Patients with Scheuermann's kyphosis may have an angular thoracic kyphosis (Figure 3.), often with accompanying compensatory lumbar lordosis and increased cervical lordosis. The position of the head is often in forward protrusion (also known as gooseneck), and the shoulders are often positioned anteriorly. Forward bending typically accentuates the kyphotic deformity, with a sharply angulated bend noted in the thoracic or thoracolumbar region. The deformity is relatively fixed, remaining during attempted hyperextension of the spine. Tightness of the hamstrings is common, but the neurologic examination is usually otherwise normal [8].

Unfortunately Wenger and Frick [8] do not describe the clinical findings of other curve patterns than thoracic Scheuermann, although the thoracolumbar and lumbar Scheuermann curve patterns are of major importance with respect to chronic low back pain in adulthood [17,19]. There seems to be less coverage of these curve patterns in the literature of Pub Med.

The degree of kyphosis on the lateral film is measured using a modified Cobb method according to Stagnara [21,22]. In addition to increased measurable roundback on the lateral view, vertebral wedging is used to clarify the diagnosis. Associated findings of scoliosis and spondylolysis can occur with Scheuermann's kyphosis, but usually are minor and do not alter treatment [8].

### Natural History

The natural history of Scheuermann's disease remains controversial. The condition tends to be symptomatic during the teenage years but often in late teenage life less pain is reported [2]. In a long term follow-up study, Sorenson noted pain in the thoracic region in 50% of patients during adolescence, with the number of symptomatic patients decreasing to 25% after skeletal maturity [8]. The pain was described as mild and 'not incapacitating'. Later authors offered a contrasting view of the symptoms of untreated Scheuermann's disease, with Bradford stating that adults with Scheuermann's kyphosis have a higher incidence of disabling back pain than the normal population [23,24].

Murray, Weinstein, and Spratt have performed a study designed to describe the natural history of Scheuermann's kyphosis [25]. They studied 67 of a group of 118 (57%) patients diagnosed by the Sorenson criteria, using physical examination, trunk strength measurements, radiography, a detailed patient questionnaire and pulmonary function testing [8]. The patients had an average kyphotic deformity of 71°, and the average follow-up was 32 years; an age-matched comparison group was used as controls. They concluded that patients with Scheuermann's kyphosis may have functional limitations, but these did not result in severe limitations due to pain, or cause major interference with their lives [8]. Yet in another paper, Lowe and Kasten stated that adults with more severe deformities (>75°) secondary to untreated Scheuermann's disease can experience severe thoracic pain secondary to degenerative spondylosis and can be significantly limited by their disease [26]. The authors allude to the greater magnitude of the deformity as a possible explanation for the life-altering pain experienced by their patients as contrasted to those reported on by Murray et al, although studies to document a direct correlation between the amount of pain and the degree of deformity are not available [8].

The common indications for treatment in Scheuermann's kyphosis are related to pain, progression of deformity and appearance. Pain is difficult to measure because of its complex subjectivity and temporal nature [8]. Most of the literature on Scheuermann's kyphosis states that pain is either present or absent, and does not provide data on how this was determined or measured [8]. The study by Murray et al. [25] is the only single attempt in the literature to objectively assess pain in this patient group. They found no statistically significant difference between the Scheuermann patients and the control group with regard to the extent that pain interfered with their lives. Although it is possible that a clinically significant difference might exist, as 38% of the Scheuermann patients had severe interference of pain with activities of daily living compared to 21% of control subjects. The kyphotic group did have significantly higher pain intensity readings and complained more frequently of pain in the thoracic region than the control group. Patients with Scheuermann's kyphosis, however, were no more likely to take medications for back pain. They were limited to only 57% of patients with Scheuermann's kyphosis and their statistics might be quite different if more patients were available for a follow-up study.

Tribus has outlined the reasons for treatment of Scheuermann's kyphosis in relation to cardiopulmonary compromise [9]. Deformity is the most common complaint of patients with Scheuermann's disease, and is typically the primary reason younger patients seek medical attention [8]. The likelihood of progression of a kyphotic curve of any given degree of severity is currently not known [27].

Studies reporting on the natural history of lumbar and thoracolumbar Scheuermann's disease have not been cited by Wenger and Frick [8] and are not available searching the Pub Med database in 2009.

### Treatment

Initial management of the patient presenting with Scheuermann's kyphosis includes documentation and assessment of the degree of deformity and/or pain, as well as an overall picture of the negative impact of the deformity on a patient's life [8]. Physical therapy (or physiotherapy) for postural improvement is often recommended, especially in central Europe, focusing on hamstring and pectoralis stretching and trunk extensor strengthening as well as improving function [28]. A good physical therapist can also assess whether there is any tendency towards increased hip flexion contracture and may work on associated lumbar lordosis [8]. There are no conclusive studies documenting improvement in kyphosis with exercises [8], although Bradford et al did note some improvement in patients with moderate degrees of deformity [29].

Scheuermann's disease in adults is regarded to be a different entity from that of the teenager for the major manifestation is pain and not aesthetic quality. The patient's occupation is rather more sedentary. The functional rehabilitation on an out-patient basis is the favoured treatment and referral for surgery or dorso-lumbar braces is rare [30].

According to Pizzutillo [31] effective interventions for adolescents with postural kyphosis include exercises to relieve lower extremity contractures and strengthen abdominal musculature, coupled with practiced normal posture in stance and in sitting. Skeletally immature patients with Scheuermann's kyphosis benefit from a similar exercise program but also require the use of a spinal orthosis. Bracing of the spine in patients with Scheuermann's kyphosis results in permanent correction of vertebral deformity, unlike bracing in patients with idiopathic scoliosis. The evaluation of children and adolescents with increased thoracic kyphosis is an important aspect of the decision process used to determine appropriate interventions [31].

### Brace treatment for hyperkyphosis

The few available studies on efficacy of brace treatment are retrospective, have different inclusion criteria and do not have control groups. In addition, as noted above, we do not yet have data available to allow us to predict which kyphotic curves are at significant risk for progression [8]. Despite these shortcomings, bracing is widely regarded as being efficacious in the treatment of Scheuermann's kyphosis in the skeletally immature patient [9,11]. Bracing has been used primarily for the treatment of deformity, with results of treatment focusing on improvement in kyphosis; the results of brace treatment for relieving pain have not yet been published [8].

**Figure 4:**
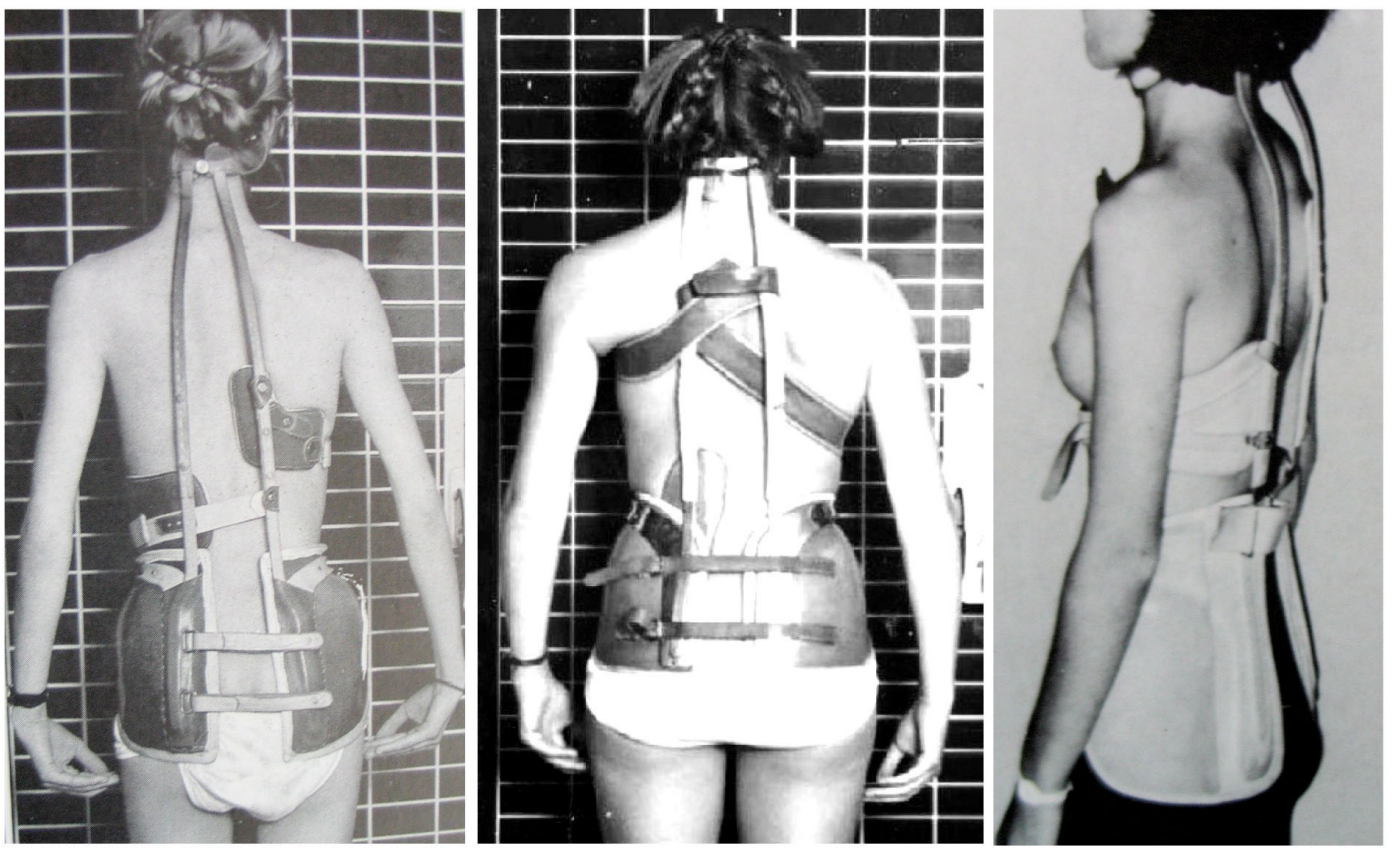
**Milwaukee braces in different patients**. These braces have been used for the treatment of patients with scoliosis in the 70es here in Germany, however for the treatment of kyphosis the Gschwend type braces have been used. Therefore no Milwaukee brace used for a patient with kyphosis is available in our data base. Nevertheless the traction in these braces is clearly visible.

The initial report of Bradford et al. on Milwaukee brace treatment (Figure 4) of Scheuermann's kyphosis in 75 patients, who had completed treatment, documented a 40% decrease in mean thoracic kyphosis and a 35% decrease in mean lumbar lordosis after an average 34 months of brace wear [29]. A later study from the same center [32] reporting on 120 of 274 patients treated with a Milwaukee brace for Scheuermann's kyphosis showed a pattern of initial correction of approximately 50% of the kyphosis (when setting the estimated normal value as 0 [33]) followed by loss of correction. Similar findings have been reported by Montgomery and Erwin [34].

Gutowski and Renshaw [35] have reported on the use of the Boston lumbar and modified Milwaukee orthoses for Scheuermann's kyphosis and abnormal juvenile round-back with an average 26-month follow-up. Out of 75 patients in their study group, 31% completely rejected the orthosis within 4 months. Compliant patients had an average improvement in kyphosis of 27% in the Boston group and 35% in the Milwaukee group, despite use of the Milwaukee brace for older patients who had greater curves [8].

The overall results of brace treatment seem reproducible [36] and promise a permanent correction of vertebral deformity, unlike bracing in patients with idiopathic scoliosis [37].

According to Lowe [38,39] brace treatment is almost always successful in patients with kyphosis between 55 degrees and 80 degrees if the diagnosis is made before skeletal maturity. Kyphosis greater than 80 degrees in the thoracic spine or 65 degrees in the thoracolumbar spine is almost never treated successfully without surgery in symptomatic patients. Surgical treatment in adolescents and young adults should be considered if there is documented progression, refractory pain, loss of sagittal balance, or neurologic deficit.

The patients treated with a brace often face problems with their relations with friends, while they reported difficulties in activities such as; getting up from bed and sleeping at night more frequently than their unaffected peers as described by Korovessis et al. [40]. As they grow older, patients develop body image related issues as they are more concerned about the future effect of the deformity on their body. As the bracing time increases, patients have much more probability than controls to develop low back pain. Girls with deformity have a higher probability than boys to get lower back pain. Individuals with larger spinal curvatures have more difficulties in bending and increased incidence of back pain than their counterparts with smaller curvatures. Psychological reasons associated mainly with relations at school and back pain are the main causes for low compliance in adolescents with spinal deformities treated with braces. Careful instructions for all individuals who will undergo brace therapy, psychological support for all patients who develop psychological reactions and physical training particularly for older girls should be recommended to increase bracing compliance [40].

A newly designed brace in the treatment of adolescent thoracic Scheuermann's kyphosis has been proposed by Riddle et al [36]. However the authors did not report on in-brace corrections.

Twenty-two children who met the roentgenographic criteria of Scheuermann's kyphosis and were compliant with treatment were followed until skeletal maturity. Sixteen patients (73%) showed non-progression of their kyphosis (nine patients demonstrated an improvement, seven patients remained unchanged), and had a mean improvement of 9 degrees (64 degrees to 55 degrees). Six patients (27%) demonstrated progression of the kyphosis and had a mean increase in their kyphosis of 9 degrees (59 degrees to 68 degrees). One patient underwent posterior spinal fusion for progressive thoracic kyphosis despite bracing. It was recommended that this brace be worn until skeletal maturity; in this study the time period was determined to be at least 16 months to induce improvement or halt progression of this disease. Flexible curves are a positive predictor of a successful outcome of bracing with the kyphosis brace. These results are comparable to previous reports in the literature describing the effectiveness of the modified Milwaukee brace in the treatment of Scheuermann's thoracic kyphosis prior to skeletal maturity, and the kyphosis brace has the advantage of concealability under normal attire [36].

**Figure 5:**
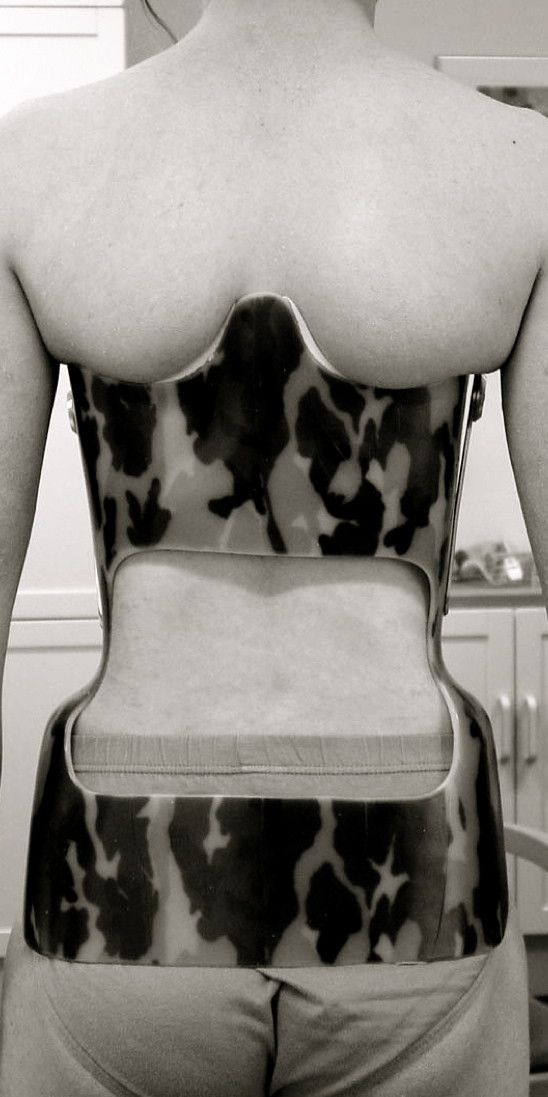
**Dorsal aspect of a Gschwend type brace as used in Germany**. Still today this kind of brace is used for the treatment of thoracic kyphosis.

Historically brace treatment in central Europe has differed, although in the 1970's the Milwaukee brace was used for the treatment of Scheuermann's kyphosis in Germany as well, but a the Gschwend brace was also used [41]. A permanent correction of kyphosis has been reported using the Gschwend-brace (Figure 5.) when the therapy started early, lasted long enough in patients with good compliance [42].

**Figure 6:**
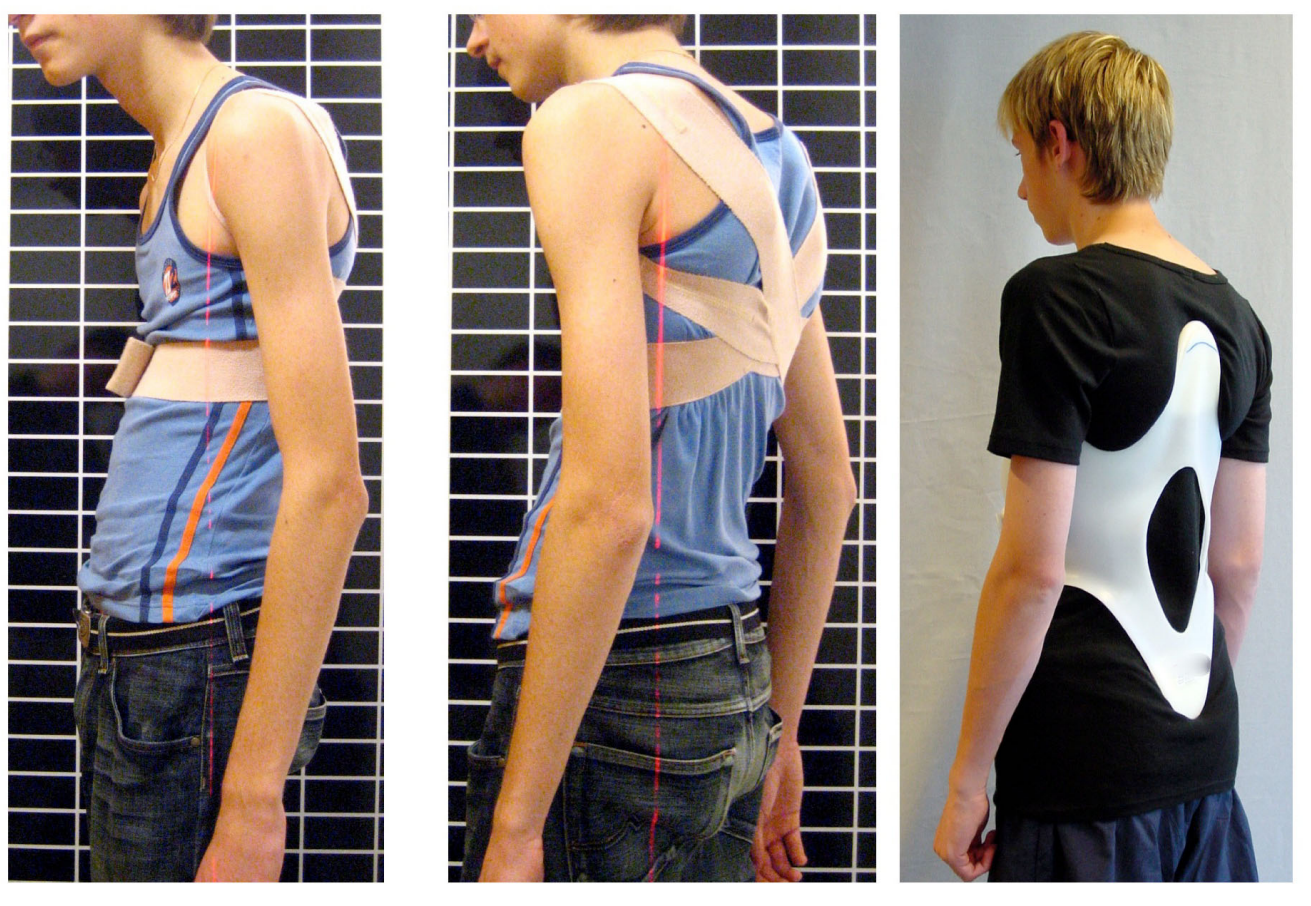
**Soft brace in the treatment of a rigid kyphosis**. Obviously no correction can be achieved using a soft brace in rigid curvatures. In the kyphologic™ brace, as can be seen on the right, a clinical correction has been achieved in the same patient.

Other bracing strategies have been attempted such as the use of a soft brace but this however has not shown to be successful (Figure 6.).

Variation of vertebral morphology in Scheuermann's kyphosis before and after orthopaedic treatment is usually measured by the entity of the curve, using Cobb's method, and by vertebral wedging. But the lack of correlation between these parameters and the clinical evolution of the deformity, lead to the possibility, that other variables may explain the reason of the kyphosis deformities before and after the treatment. In this group of alterations the inclination of anterior and posterior walls, that express the trapezoid deformity of the vertebra, seem to be more reliable indicators of curve response to orthopaedic treatment [43].

**Figure 7:**
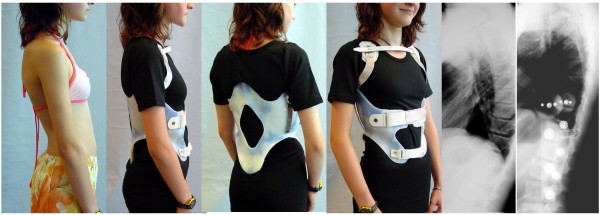
**Patient with rigid kyphosis in a kyphologic™ brace**. Patient with rigid kyphosis without and in a kyphologic™ brace. The in-brace correction is good and after the first 6 weeks of treatment the curve was flexible again so as to allow to reduce brace wearing time to 16 hrs./day.

**Figure 8:**
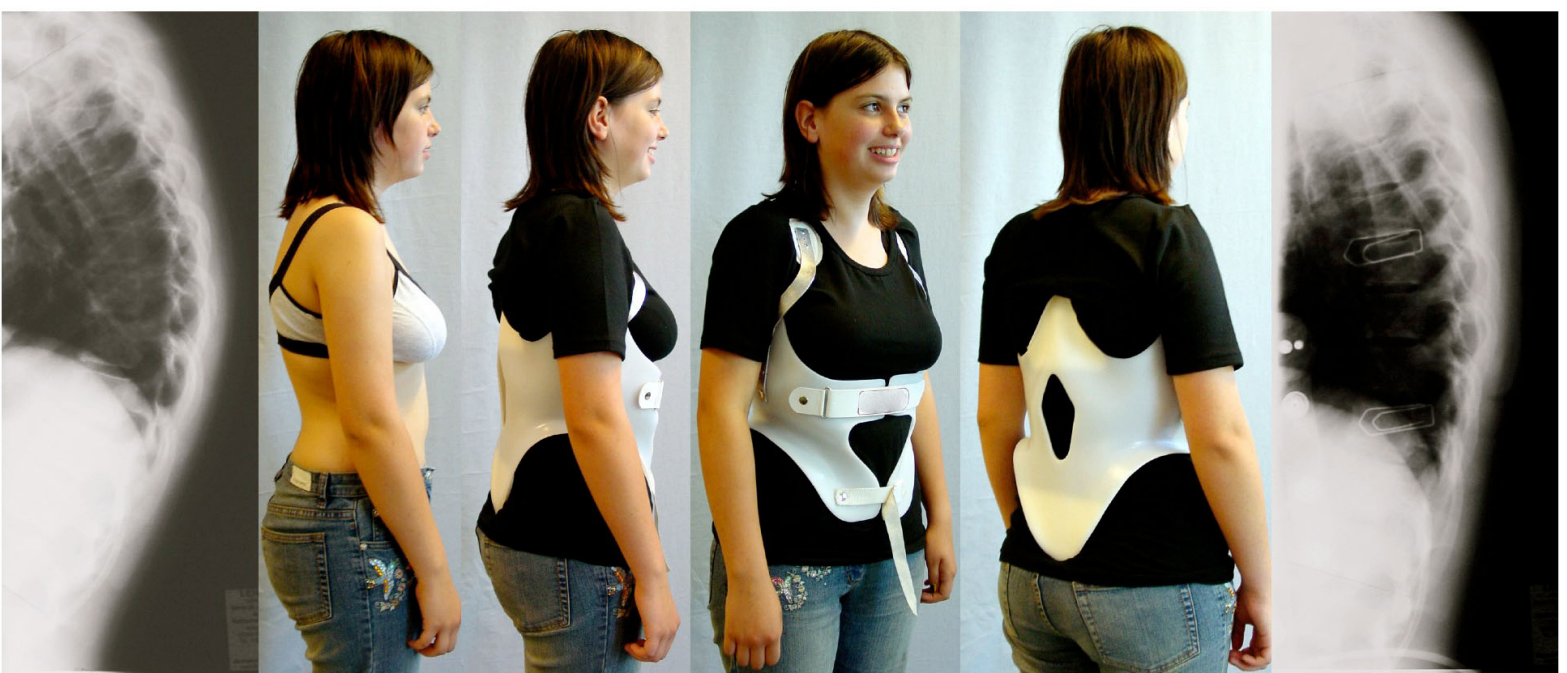
**Another patient with rigid kyphosis in a kyphologic™ brace**. Patient with rigid kyphosis without and in a kyphologic™ brace. The in-brace correction is good and after the first 6 weeks of treatment - as in most of the patients treated with this brace - the curve was flexible again so as to allow to reduce brace wearing time to 16 hrs./day as well.

Results of modern braces used for the treatment of kyphosis have also been documented in a recent textbook on spinal deformities [44]. In order to improve the patient's quality of life whilst wearing the brace and by that improving compliance as well, attempts have been made to reduce the material of the current braces as applied without losing the in-brace correction desired [45]. This brace, known as the kyphologic™ brace, was developed in 2005 and meanwhile is applied to a number of kyphosis patients (Figure 7 and 8.).

Little is known about the in-brace correction effects of braces used for the treatment of kyperkyphosis. While Bradford et al. [24,29,32] have found their attempts effective, treating Scheuermann's kyphosis with Milwaukee braces, they did not widely report on in-brace corrections. According to White and Panjabi [46] it seems the appropriate approach to try to correct a curvature of > 50° with the help of distraction forces, patient comfort however was compromised in the Milwaukee brace. To avoid this limitation in Germany braces generally prescribed for hyperkyphosis treatment use transverse correction forces only without any neck ring. Efforts to reduce brace material have resulted in a special bracing design called kyphologic™ brace as described above. The aim of this paper is to study the possible in-brace corrections which have been achieved with this new brace design in comparison to others in related research.

## Materials and methods

56 adolescents with the diagnosis of a thoracic Scheuermann's kyphosis or a thoracic idiopathic kyphosis (22 girls and 34 boys) with an average age of 14 years (12-17 yrs.) have been treated with the kyphologic™ brace and physiotherapy of variable intensity between May 2007 and October 2008.

**Figure 9:**
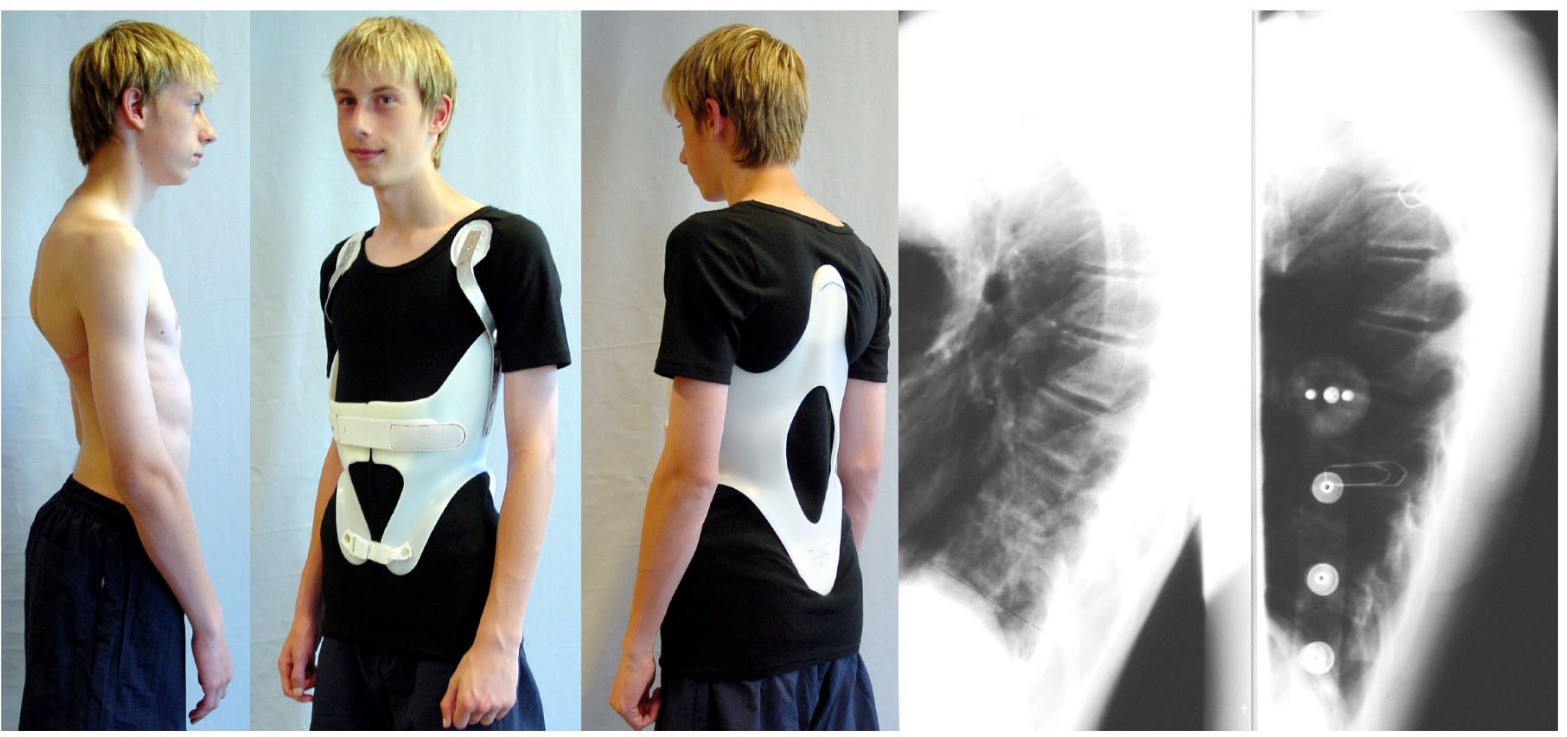
**A good in-brace correction in a patient with rigid kyphosis in a kyphologic™ brace**. Patient with rigid kyphosis without and in a kyphologic™ brace. The in-brace correction is > 20° and therefore can be regarded as being sufficient for an end-result improvement when compliance can be achieved.

**Figure 10:**
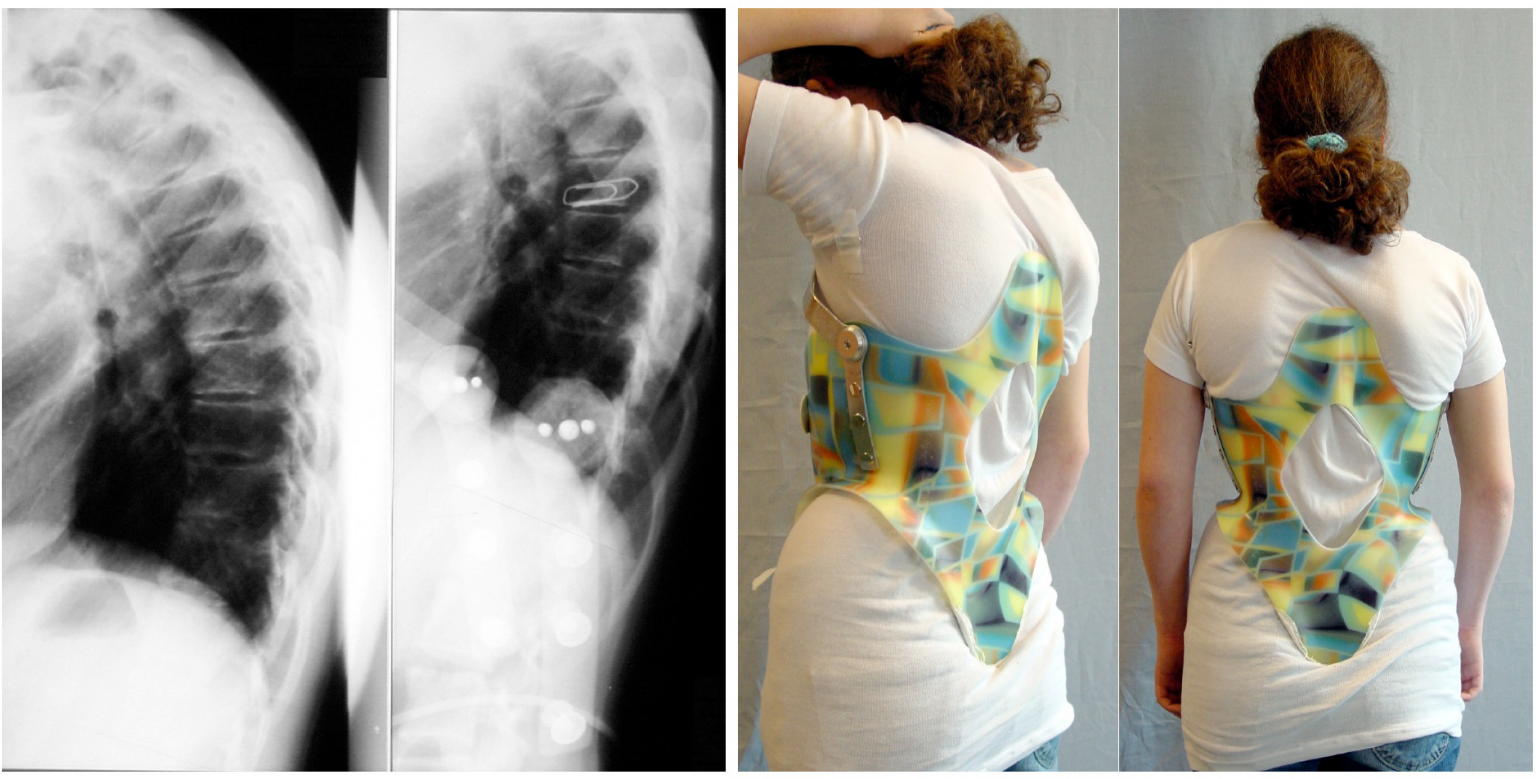
**Another patient with good in-brace correction in a kyphologic™ brace**. Patient with rigid kyphosis in a kyphologic™ brace. The in-brace correction is > 20° in this patient as well.

**Figure 11:**
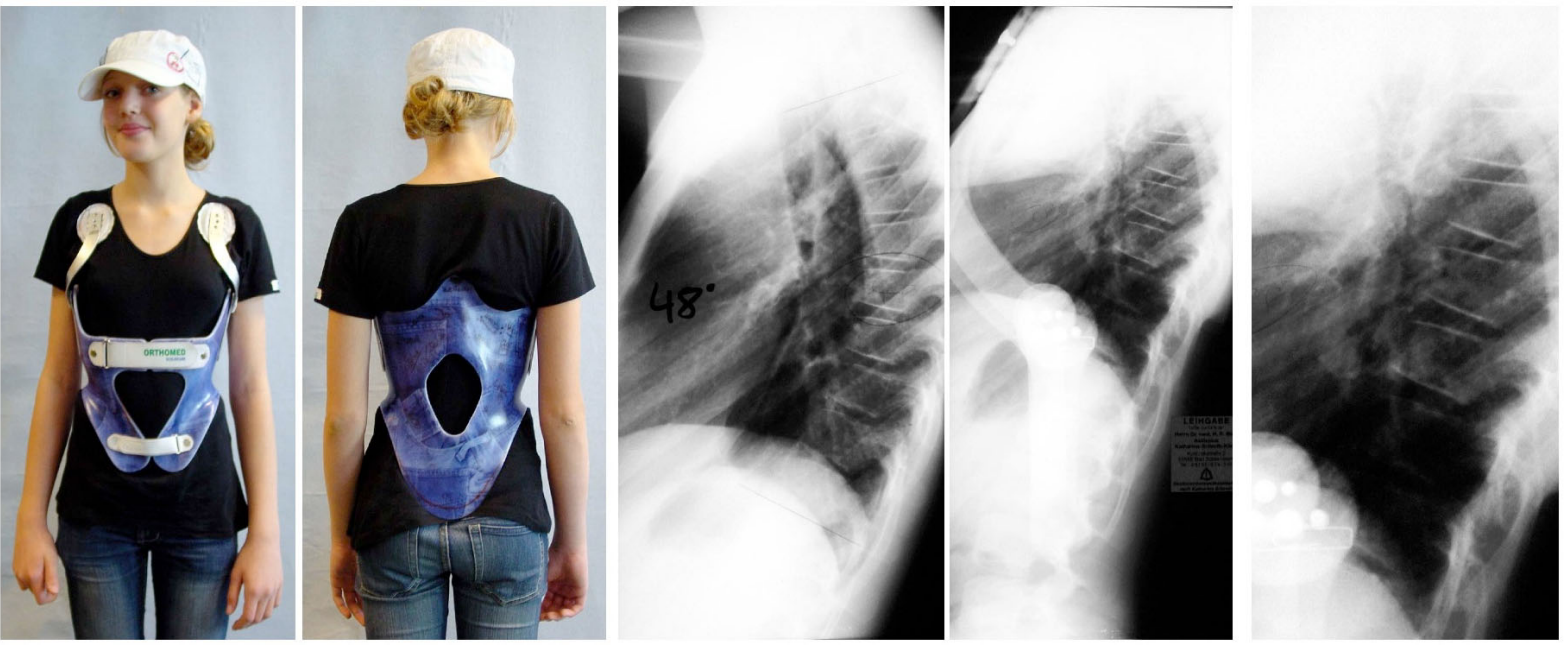
**A good in-brace correction in a patient with rigid kyphosis in a kyphologic™ brace showing a release of the anterior growth plates**. Patient with rigid kyphosis in a kyphologic™ brace. The in-brace correction is > 20° and on the right picture the opening of the intervertebral spaces is visible. Therefore we can expect an end result correction when the brace is worn, because the ventral growth plates have been released from pressure.

The kyphologic™ brace, like the Gschwend type braces [41], uses two 3-point pressure systems. The first of the two 3-point pressure systems includes the sacral pad dorsally, the subpectoral rib pressure area ventrally and the thoracic (apical) pressure area dorsally. The second 3-point system includes the subpectoral rib pressure area ventrally, the thoracic (apical) pressure area dorsally and the two pads redressing the shoulder girdle ventrally aiming at the cavity of the coracoid process (Figure 7, 8, 9, 10 and 11). The upper closure strap is directed at applying pressure on the lower ribs so as to inhibit protrusion of the lower ribs, while the lower closure is simply for pelvic fixation.

Like the Gschwend type braces [41] the kyphologic™ brace is designed for thoracic curve patterns and therefore the subject selection criterion was a rigid thoracic kyphosis.

To compare the in-brace corrections of the kyphologic™ brace with that of other braces already described in international literature, the Stagnara kyphosis angle without brace, the in-brace kyphosis angle (after 6 weeks of brace treatment) and the differences between the two angles have been recorded together with the patient's general data (Name, sex and age).

The Stagnara angle in thoracic kyphoses was measured from T4 (next upper vertebra visible, when T4 was not clearly visible) to the lower end vertebra of the curve.

Average Stagnara angle was 55.6° (43-80). In-brace correction as measured on a lateral X-ray of the thoracic spine in standing position whilst in the brace was recorded and compared to the initial angle on a lateral X-ray with the help of the t-test. The X-rays were taken in standardised standing position. To estimate the in-brace correction in percentages the total Stagnara angle was valued as 100% and therefore the described percentage of in-brace correction was related to the total Stagnara angle.

The problem with the calculation of the in-brace correction is the wide range of norm-values, making it impossible to describe and calculate the in-brace correction as achieved in a standardised way. Authors reporting on in-brace corrections in kyphosis treatment have solved the problem by determining the kyphosis angle of 25° as equalling 0 value [29,33].

To make our results comparable to the other few studies published on that topic, the kyphosis angle of 25° was set to 0 for our sample also, so as to achieve a norm value adapted (NVA) in-brace correction.

## Results

Average Stagnara angle in the brace was 39°. The average in-brace correction was 16.5° (1-40°). Average in-brace correction in percentage of the initial value was 36%. The differences were significant in the t-test (t = 5,31, p < 0,001). There was no correlation between the percentage of in-brace correction and the age of the patient (r = -0,035), but a high significant correlation between the percentage of in-brace correction and the initial Stagnara angle (r = -0,62; p < .01).

The norm-value adapted (NVA) in-brace correction was 54.1%.

## Discussion

If we assume that outcome of brace treatment positively correlates with in-brace correction the treatment should start before the curvature angle exceeds 50 - 55° in patients that are continuing to grow. In scoliosis bracing an average in-brace correction of > 15° predicts an end result correction [32]. At average with this new brace we have achieved > 15° also in hyperkyphosis treatment. Therefore we estimate to achieve a favourable outcome using this brace type when compliance can be gained.

The kyphologic™ brace leads to in-brace corrections comparable to those of the Milwaukee brace, which have been shown to lead to a beneficial outcome in the long-term [32-39].

These results have been achieved with much less brace material and less compromise of quality of life of the patients treated (Figure 9, 10, 11.). Therefore the Milwaukee brace, although leading to beneficial outcomes, when compared with newer bracing approaches has been shown to be less effective.

The Gschwend type braces, with full pelvic coating, in kyphosis treatment seem no longer necessary. When trying to correct a kyphosis, lateral pelvic support does not seem to be necessary, when the counter forces acting are focused specifically upon the sagittal plane.

**Figure 12:**
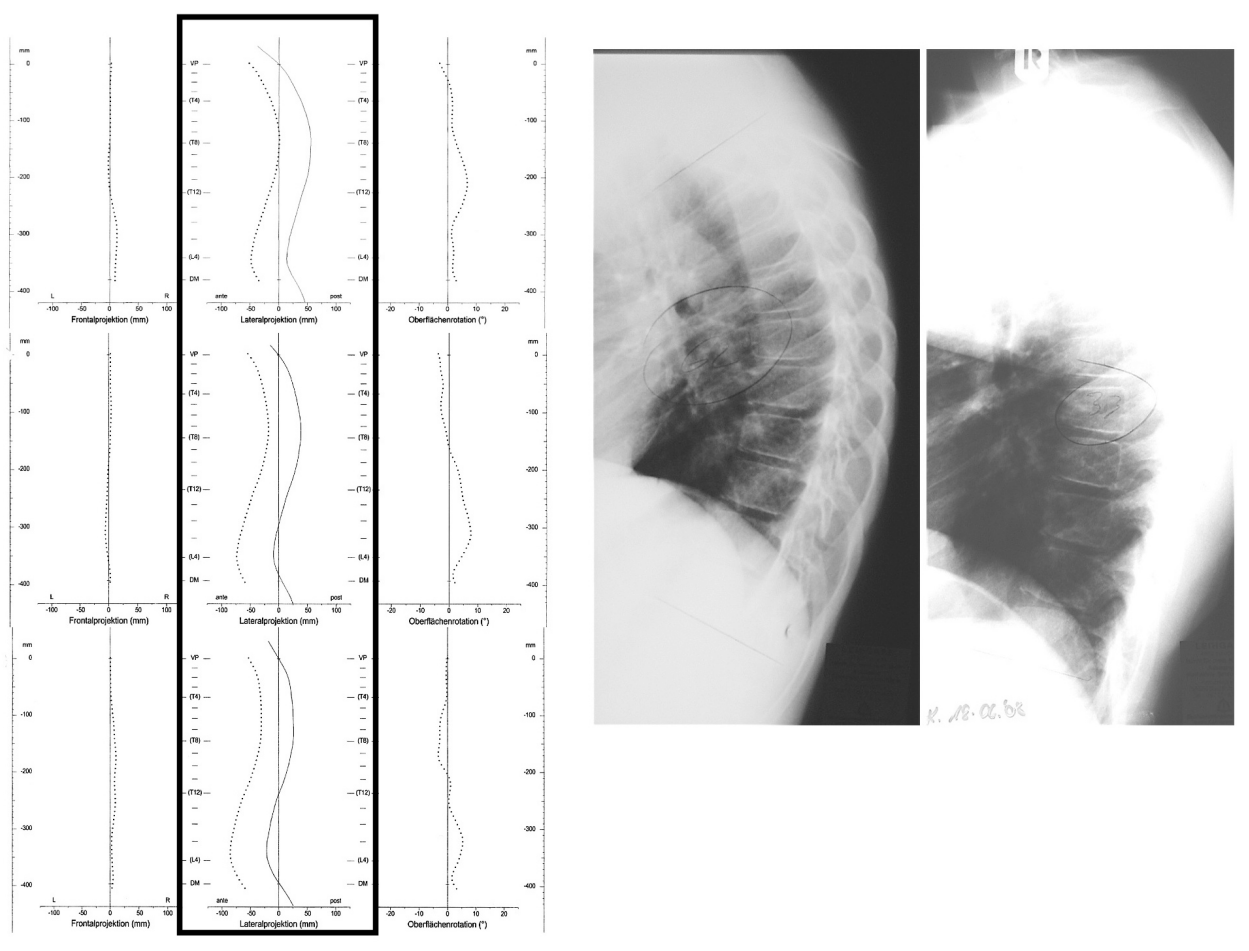
**Correction achieved within 15 months of treatment**. The in-brace correction has been 30° and before a new brace has been made the curve was 33° whereas initially we have measured > 60°. The sagittal surface topography reconstruction can be seen in the black frame on the left: Upper picture at the start of treatment, middle picture after 6 months of treatment and lower picture after 15 months. A straightening of the spine in sagittal plane is clearly visible.

A few patients from this study with follow-up periods of more than one year have been registered. One boy with an in-brace correction of 30 degrees reached an intermediate correction of 30 degrees (without the brace on) before it was necessary to construct a new brace (Figure 12).

**Figure 13:**
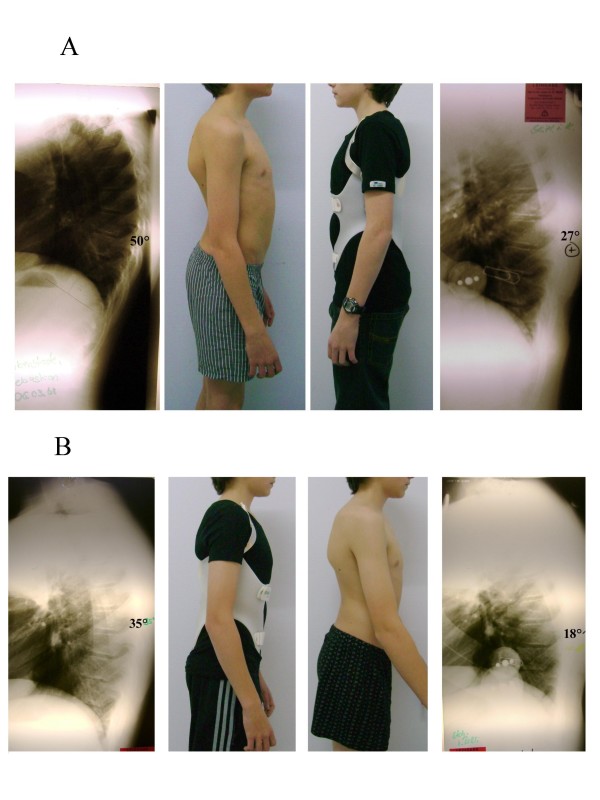
**In-brace correction in two subsequent braces of one patient with rigid kyphosis**. A. In-brace correction in the first brace of a patient with rigid kyphosis: The correction was from 50° initially to 27° in the first brace. B. In-brace correction in the second brace of the patient from figure 13 A: In the second brace the curve has been corrected from 35° to 18° and the curve was no more stiff. However as there was still growth left we adjusted a new brace for 8-12 hrs./day.

Another patient is demonstrated on Figure 13 with an improved curve after 9 months of treatment (16 hrs per day) and continued to improve his curvature correction.

Findings like this lead us to the presumption, that final corrections 2 years after weaning should be possible. Nevertheless, a prospective follow-up study seems desirable before final conclusions can be drawn.

**Figure 14:**
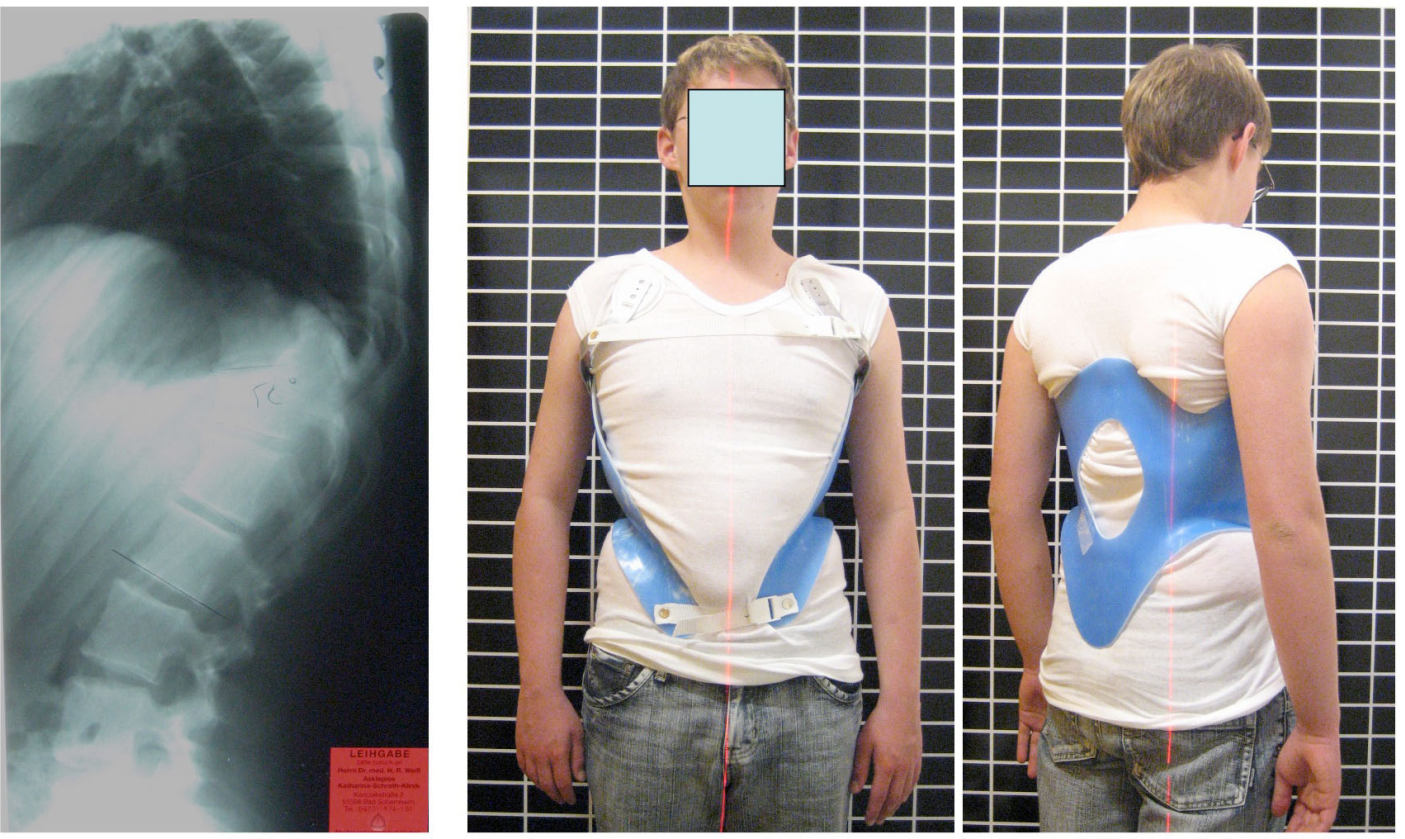
**kyphologic™ brace model for low thoracic or thoracolumbar kyphosis**. The correction effects in low thoracic and thoracolumbar curve patterns were far from being satisfying. Therefore we will not offer this kind of braces any longer.

**Figure 15:**
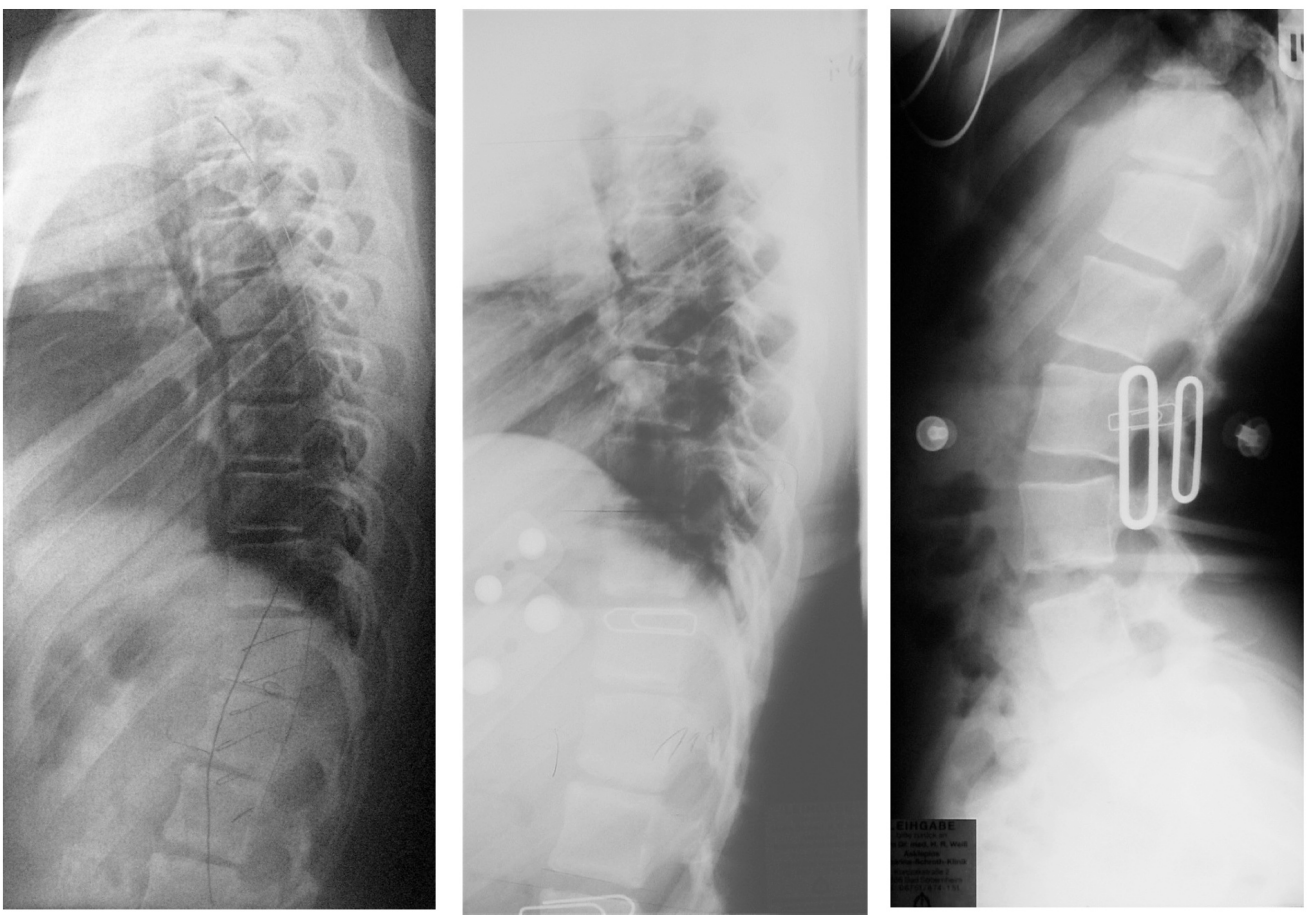
**Lateral X-rays of a patient with thoracolumbar kyphosis**. Left: lateral X-ray without brace, middle: lateral X-ray in the kyphologic™ brace model for thoracolumbar kyphosis without any significant effect. Right: In the physio-logic™ brace at least the lumbar lordosis is restored giving the sagittal view a more physiological pattern.

There are other curve patterns than thoracic ones. We distinguish between thoracic, thoracolumbar and lumbar patterns of hyperkyphosis or Scheuermann's desease. The kyphologic™ brace as it is described is indicated for the treatment of thoracic kyphosis only. We have been trying to develop a kyphologic™ brace for thoracolumbar kyphosis patterns (Figure 14.) but the results achieved were not comparable to those we were able to achieve in thoracic kyphosis (Figure 15.). Hence why the treatment of thoracolumbar curves with the physio-logic™ brace is aimed at a reduction of lumbar kyphosis in order to allow a restoration of lumbar lordosis and to allow a restoration of a physiological sagittal profile of the spine as well. This does not lead to major correction effects, as it simply shifts the kyphosis cranially (curvature phase shift). With respect to the fact that the loss of lumbar lordosis is not favorable in the long-term [15,16], pain prevention seems more important than cosmesis in these curve patterns.

**Figure 16:**
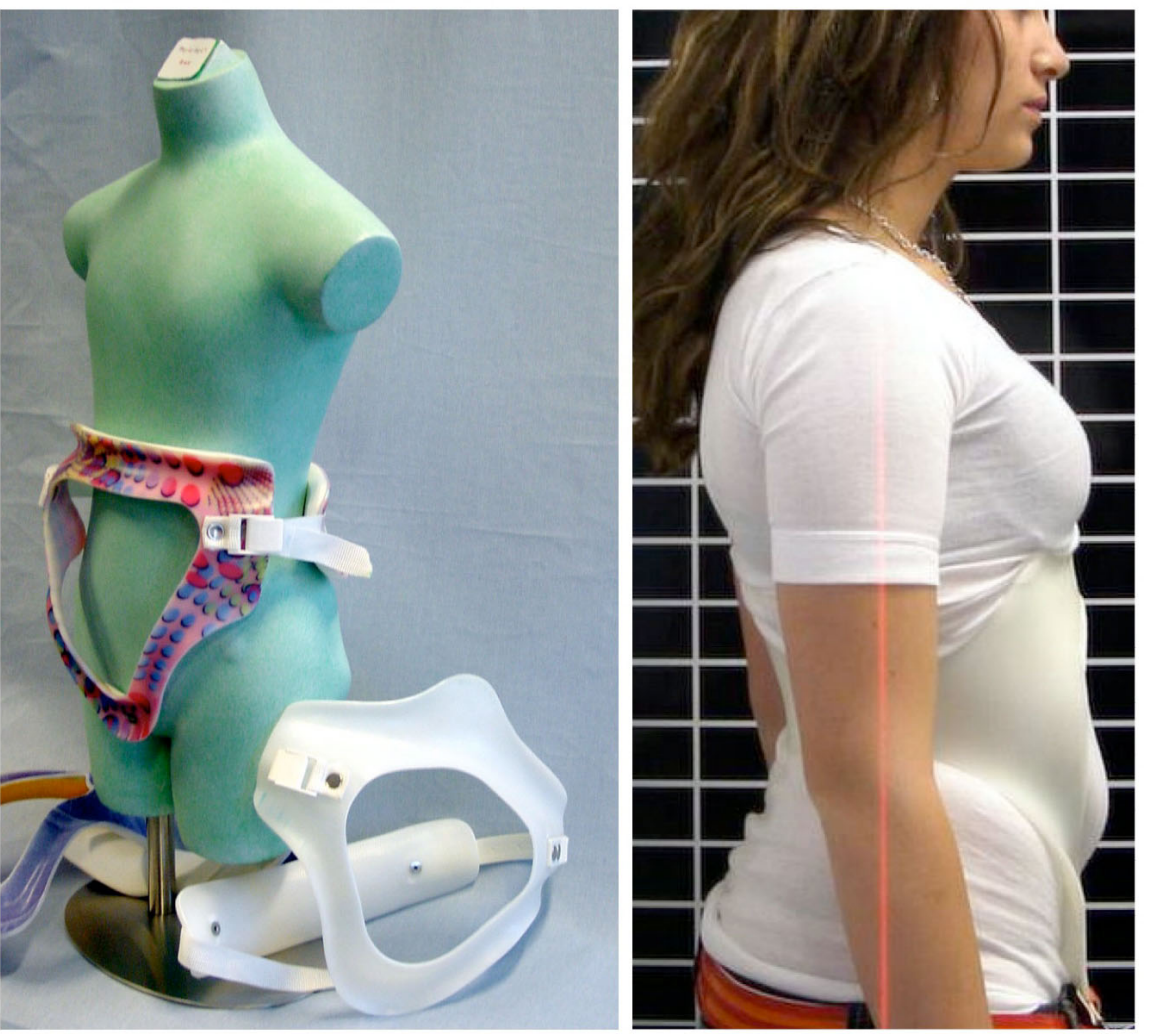
**physio-logic™ braces of the first series from 2004**. The physio-logic™ brace aims at a restoration of the lumbar lordosis with a lordosis apex at L2 or L2/3 level.

**Figure 17:**
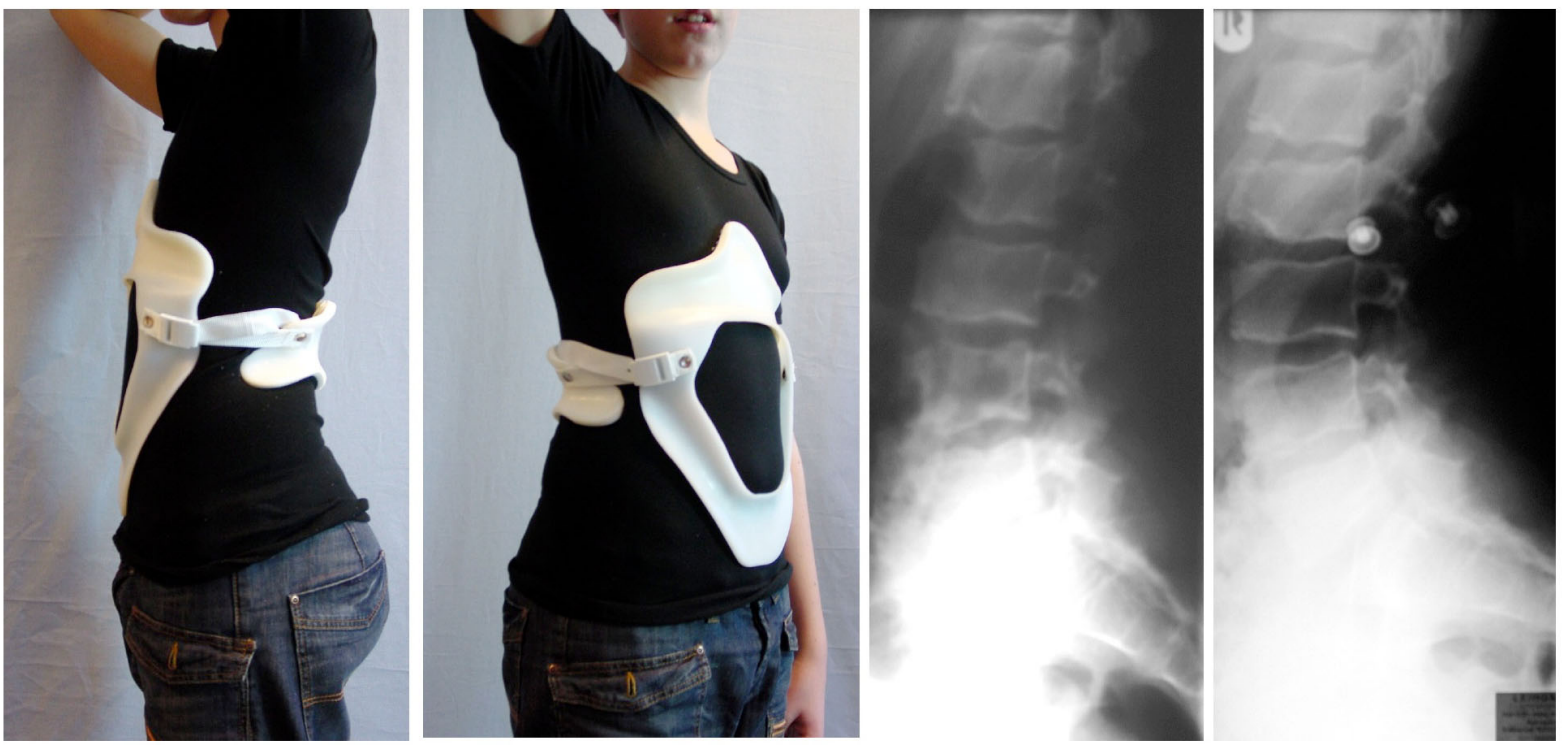
**Patient with lumbar Scheuermann in the physio-logic™ brace**. Improvement of lumbar lordosis is visible. Due to curve stiffness the in-brace correction seems moderate, however clinically the brace effect is visible as well.

Lumbar kyphoses are treated with the physio-logic™ brace (Figure 16 and 17.) for the same reasons. In-brace corrections for this rare pattern of Scheuermann desease, however have not yet been investigated.

**Figure 18:**
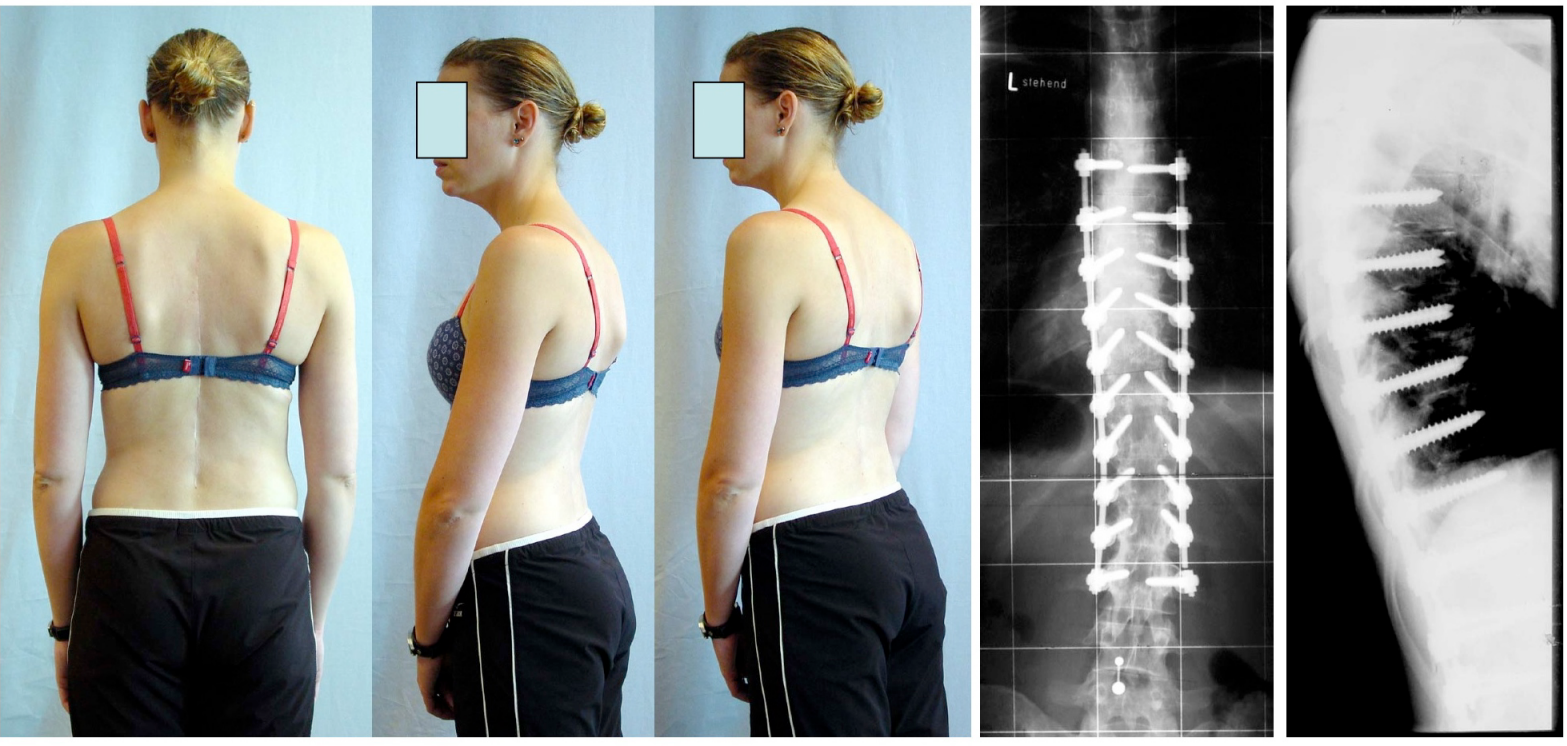
**Patient operated for a severe thoracic kyphosis**. The patient is satisfied with respect to clinical (cosmetic) appearence and has no pain in the region of the rod fracture (lower right). However she suffers from pains in the junctional zones and has daily pains in the lower back below the fusion area and above the fused area in the lower neck as well. This pain is increasing during the years.

As previously reported [8], surgical interventions (Figure 18) come with increased risks. Nevertheless recent publications seem to support surgery [47,48]. As there is a consensus, that surgery is rarely necessary because conservative management is highly effective [8], it is necessary to improve the conservative standards of treatment. To improve the compliance for conservative treatment and therefore developing a bracing design with less materials but promising in-brace correction seems a step towards improving final outcomes.

There is little literature available in Pub Med about the current concepts of bracing for hyperkyphosis. Therefore it was necessary to research this area in order to stimulate debate in this topic, which has been widely neglected in the last decade. It was the desire of the authors to use this paper as a springboard for future research. With respect to in-brace correction as other braces used successfully in the last decade, the brace presented within this paper has less of an impact on quality of life of the patients and uses less material in its creation.

## Conclusion

Conservative treatment of Scheuermann's hyperkyphosis in international literature is generally regarded as an effective approach. Physiotherapy and bracing are the first line treatments for this condition. Due to its benign character in-patient rehabilitation rarely seems necessary.

An average in-brace correction of > 15° as was achieved with the help of the kyphologic™ brace seems to predict a favourable outcome.

The kyphologic™ brace leads to in-brace corrections comparable to those of the Milwaukee brace, which has previously been shown to provide beneficial outcomes in the long-term treatment of patients with Scheuermann's hyperkyphosis.

A prospective follow-up study seems desirable before final conclusions can be drawn.

Future studies should focus more on thoracolumbar and lumbar curve patterns, because these patterns may predict chronic low back pain in adulthood with reduced quality of life of the patients and high costs with respect to medical care and occupational sickness leave.

Surgery according to international literature is rarely necessary in this condition.

## Competing interests

HRW is consultant of Koob GmbH, Abtweiler, Germany; DT and SB declare that they have no competing interests.

## Authors' contributions

HRW patient acquisition, study design, evaluation, manuscript writing, research of databases and figures. DT manuscript writing, copyediting and proof check. SB establishing the study data base, documentation. All authors read and approved the final manuscript.
